# Triprolidinium dipicrate

**DOI:** 10.1107/S160053681103457X

**Published:** 2011-08-27

**Authors:** A. S. Dayananda, Jerry P. Jasinski, James A. Golen, H. S. Yathirajan, C. R. Raju

**Affiliations:** a Department of Studies in Chemistry, University of Mysore, Manasagangotri, Mysore 570 006, India; b Department of Chemistry, Keene State College, 229 Main Street, Keene, NH 03435-2001, USA; c Department of Chemistry, PES College of Science, Mandya 571 401, India

## Abstract

In the tripodinium cation of the title compound {systematic name: 2-[(*E*)-1-(4-methyl­phen­yl)-3-(pyrrolidin-1-ium-1-yl)prop-1-en­yl]pyridinium bis­(2,4,6-trinitro­phenolate)}, C_19_H_24_N_2_^+^·2C_6_H_2_N_3_O_7_^−^, the N atoms on both the pyrrolidine and pyridinium groups are protonated. The pyrrolidine group adopts a slightly distorted envelope configuration. Strong N—H⋯O cation–anion hydrogen bonds and weak inter­molecular N—H⋯O inter­actions link the dication and two anions. In both picrate anions, the nitro groups display rotational disorder over two orientations in a 0.605 (6):0.395 (6) ratio. The crystal packing also features weak inter­molecular π–π [centroid–centroid distance = 3.8036 (14) Å] and C—H⋯O inter­actions.

## Related literature

For anti­cholinergic properties, see: Salunga *et al.* (1996 ▶). For related structures, see: James & Williams (1971 ▶, 1974 ▶); Parvez & Sabir (1997 ▶). For puckering paramerers, see: Cremer & Pople (1975 ▶). For bond lengths, see: Allen *et al.* (1987 ▶).
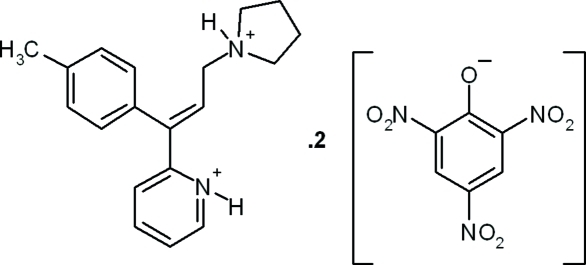


## Experimental

### 

#### Crystal data

C_19_H_24_N_2_^2+^·2C_6_H_2_N_3_O_7_^−^
*M* *_r_* = 736.61
Monoclinic, 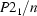

*a* = 15.0542 (7) Å
*b* = 12.7489 (5) Å
*c* = 17.1446 (7) Å
β = 100.218 (4)°
*V* = 3238.3 (2) Å^3^
*Z* = 4
Mo *K*α radiation
μ = 0.12 mm^−1^
*T* = 173 K
0.34 × 0.23 × 0.21 mm

#### Data collection

Oxford Diffraction Xcalibur Eos Gemini diffractometer
Absorption correction: multi-scan (*CrysAlis RED*; Oxford Diffraction, 2010 ▶) *T*_min_ = 0.960, *T*_max_ = 0.975
32796 measured reflections
8355 independent reflections
6144 reflections with *I* > 2σ(*I*)
*R*_int_ = 0.027

#### Refinement

*R*[*F* ^2^ > 2σ(*F* ^2^)] = 0.067
*wR*(*F* ^2^) = 0.192
*S* = 1.02
8355 reflections
486 parameters
14 restraints
H atoms treated by a mixture of independent and constrained refinement
Δρ_max_ = 0.63 e Å^−3^
Δρ_min_ = −0.42 e Å^−3^

### 

Data collection: *CrysAlis PRO* (Oxford Diffraction, 2010 ▶); cell refinement: *CrysAlis PRO*; data reduction: *CrysAlis RED* (Oxford Diffraction, 2010 ▶); program(s) used to solve structure: *SHELXS97* (Sheldrick, 2008 ▶); program(s) used to refine structure: *SHELXL97* (Sheldrick, 2008 ▶); molecular graphics: *SHELXTL* (Sheldrick, 2008 ▶); software used to prepare material for publication: *SHELXTL*.

## Supplementary Material

Crystal structure: contains datablock(s) global, I. DOI: 10.1107/S160053681103457X/xu5298sup1.cif

Structure factors: contains datablock(s) I. DOI: 10.1107/S160053681103457X/xu5298Isup2.hkl

Supplementary material file. DOI: 10.1107/S160053681103457X/xu5298Isup3.cml

Additional supplementary materials:  crystallographic information; 3D view; checkCIF report

## Figures and Tables

**Table 1:** Hydrogen-bond geometry (Å, °)

*D*—H⋯*A*	*D*—H	H⋯*A*	*D*⋯*A*	*D*—H⋯*A*
N1—H1N⋯O10	0.85 (2)	1.89 (2)	2.701 (2)	157 (2)
N1—H1N⋯O8	0.85 (2)	2.44 (2)	3.053 (7)	129 (2)
N1—H1N⋯O8*A*	0.85 (2)	2.57 (2)	3.173 (10)	128 (2)
N2—H2N⋯O3	0.86 (2)	1.85 (2)	2.648 (2)	153 (2)
C31—H31*A*⋯O11^i^	0.95	2.58	3.520 (3)	170
C9—H9*A*⋯O11	0.95	2.47	3.174 (3)	131
C5—H5*B*⋯O3^ii^	0.99	2.44	3.362 (4)	154
C5—H5*A*⋯O13*A*^iii^	0.99	2.47	3.399 (6)	157
C4—H4*A*⋯O14*A*^iv^	0.99	2.33	3.116 (7)	136

Symmetry codes: (i) 
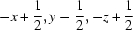
; (ii) 
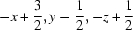
; (iii) 
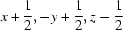
; (iv) 
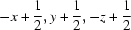
.

**Fig. 1.**
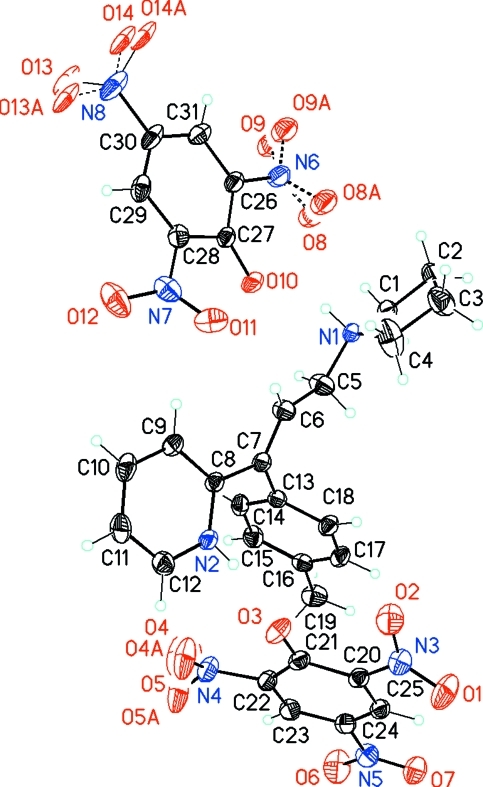
Molecular structure of the title compound, showing the atom-labeling scheme and 30% probability displacement ellipsoids.

**Fig. 2.**
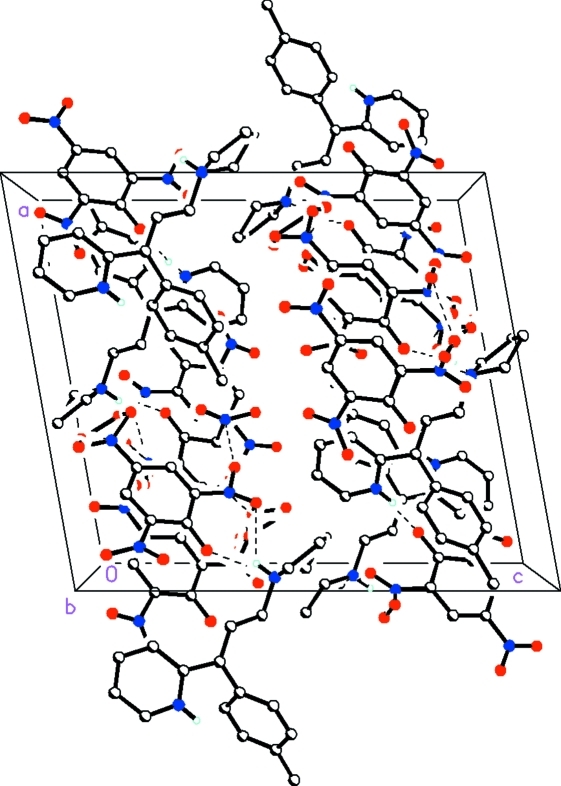
Packing diagram of the title compound, viewed down the b axis. Dashed lined indicate N—H···O hydrogen bonds.

## supplementary crystallographic information

### Comment

Triprolidine, is a histamine H1 antagonist that competes with histamine for the normal H1-receptor sites on effector cells of the gastrointestinal tract, blood vessels and respiratory tract. Triprolidine has anticholinergic properties and is used to combat the symptoms associated with allergies and is sometimes combined with other cold medications designed to provide general relief for flu-like symptoms (Salunga *et al.*, 1996). The crystal structures of triprolidine hydrochloride (James & Williams, 1971), triprolidine hydrochloride monohydrate (James & Williams, 1974) and triprolidine tetrachlorocuprate (II) (Parvez & Sabir, 1997) have been reported. In view of the importance of the title compound, this paper reports the crystal structure of (I), C_19_H_24_N_2_^+^. C_12_H_4_N_6_O_14_^-^.

In the tripodinium cation of the title compound [systematic name: 2-[(E)-1-(4-methylphenyl)-3-pyrrolidin-1-yl-prop-1-enyl]pyridinium bis(2,4,6-trinitrophenolate)], C_19_H_24_N_2_^+^. C_12_H_4_N_6_O_14_^-^., the N atoms on the pyrrolidine and pyridinium groups are protonated (Fig.1). The pyrrolidine group adopts a slightly distorted envelope configuration with puckering parametes Q = 0.288 (3)Å; φ = 32.2 (6)° (Cremer & Pople, 1975). Strong N—H···O cation-anion hydrogen bonds and weak N—H···O intermolecular interactions (Table 1) link the dication and two anions (Fig. 2).  Bond lengths are in normal ranges (Allen *et al.*, 1987). In both picrate anions, three of the nitro groups are rotationally disordered over two positions in a ratio of 0.605 (6): 0.395 (6) [O4A & O5A (0.395 (6), O4 & O5 (0.605 (6); O8A & O9A (0.395 (6), O8 & O9 (0.605 (6); O13A & O14A (0.605 (6), O13 & O14 (0.395 (6)].  The crystal packing is stabilized by weak intermolecular π–π [Cg4—Cg5 (3/2-x, 1/2+y, 1/2-z) centroid distances = 3.8036 (14)Å; Cg4 = C26—C31 & Cg5 = C20—C25] and C—H···O interactions forming chains along the *a* axis.

### Experimental

Triprolidine hydrochloride (3.15 g, 0.01 mol) was dissolved in 10 ml of methanol and picric acid (2.29 g, 0.01 mol) was dissolved in 10 ml of methanol. Both the solutions were mixed and stirred in a beaker at 333 K for 30 minutes. The mixture was kept aside for three days at room temperature. The formed salt was filtered & dried in a vaccum desiccator over phosphorous pentoxide. The compound was recrystallized from dimethyl sulphoxide by slow evaporation (m.p: 466-468 K).

### Refinement

The oxygen atoms of three nitro groups on picrate cations are disordered over two positions in a ratio of 0.605 (6):0.395 (6).

H1N and H2N were located by a Fourier map and refined isotropically. All of the remaining H atoms were placed in their calculated positions and then refined using the riding model with C–H lengths of 0.95 Å (CH), 0.99 Å (CH_2_) or 0.98 Å (CH_3_).  The isotropic displacement parameters for these atoms were set to 1.2 (CH, CH_2_) or 1.5 (CH_3_) times *U*_eq_ of the parent atom.

### Crystal data

**Table:**

C_19_H_24_N_2_^2+^·2C_6_H_2_N_3_O_7_^−^	*F*(000) = 1528
*M**_r_* = 736.61	*D*_x_ = 1.511 Mg m^−^^3^
Monoclinic, *P*2_1_/*n*	Mo *K*α radiation, λ = 0.71073 Å
Hall symbol: -P 2yn	Cell parameters from 11024 reflections
*a* = 15.0542 (7) Å	θ = 3.2–32.3°
*b* = 12.7489 (5) Å	µ = 0.12 mm^−^^1^
*c* = 17.1446 (7) Å	*T* = 173 K
β = 100.218 (4)°	Block, colorless
*V* = 3238.3 (2) Å^3^	0.34 × 0.23 × 0.21 mm
*Z* = 4	

### Data collection

**Table:**

Oxford Diffraction Xcalibur Eos Gemini diffractometer	8355 independent reflections
Radiation source: Enhance (Mo) X-ray Source	6144 reflections with *I* > 2σ(*I*)
graphite	*R*_int_ = 0.027
Detector resolution: 16.1500 pixels mm^-1^	θ_max_ = 28.7°, θ_min_ = 3.2°
ω scans	*h* = −20→20
Absorption correction: multi-scan (*CrysAlis RED*; Oxford Diffraction, 2010)	*k* = −17→17
*T*_min_ = 0.960, *T*_max_ = 0.975	*l* = −22→23
32796 measured reflections	

### Refinement

**Table:**

Refinement on *F*^2^	Primary atom site location: structure-invariant direct methods
Least-squares matrix: full	Secondary atom site location: difference Fourier map
*R*[*F*^2^ > 2σ(*F*^2^)] = 0.067	Hydrogen site location: inferred from neighbouring sites
*wR*(*F*^2^) = 0.192	H atoms treated by a mixture of independent and constrained refinement
*S* = 1.02	*w* = 1/[σ^2^(*F*_o_^2^) + (0.087*P*)^2^ + 2.0922*P*] where *P* = (*F*_o_^2^ + 2*F*_c_^2^)/3
8355 reflections	(Δ/σ)_max_ = 0.019
486 parameters	Δρ_max_ = 0.63 e Å^−^^3^
14 restraints	Δρ_min_ = −0.42 e Å^−^^3^

### Special details

**Table:**

Geometry. All esds (except the esd in the dihedral angle between two l.s. planes) are estimated using the full covariance matrix. The cell esds are taken into account individually in the estimation of esds in distances, angles and torsion angles; correlations between esds in cell parameters are only used when they are defined by crystal symmetry. An approximate (isotropic) treatment of cell esds is used for estimating esds involving l.s. planes.
Refinement. Refinement of *F*^2^ against ALL reflections. The weighted *R*-factor *wR* and goodness of fit *S* are based on *F*^2^, conventional *R*-factors *R* are based on *F*, with *F* set to zero for negative *F*^2^. The threshold expression of *F*^2^ > σ(*F*^2^) is used only for calculating *R*-factors(gt) *etc*. and is not relevant to the choice of reflections for refinement. *R*-factors based on *F*^2^ are statistically about twice as large as those based on *F*, and *R*- factors based on ALL data will be even larger.

### Fractional atomic coordinates and isotropic or equivalent isotropic displacement parameters (Å^2^)

**Table:**

	*x*	*y*	*z*	*U*_iso_*/*U*_eq_	Occ. (<1)
O1	0.92233 (17)	0.70372 (18)	0.04819 (15)	0.0835 (7)	
O2	0.82310 (14)	0.65125 (19)	0.11427 (12)	0.0733 (6)	
O3	0.88982 (12)	0.56044 (14)	0.25150 (11)	0.0561 (4)	
O4A	0.9819 (13)	0.4781 (13)	0.3699 (8)	0.0957 (11)	0.395 (6)
O4	0.9885 (8)	0.4552 (8)	0.3767 (5)	0.0957 (11)	0.605 (6)
O5A	1.0956 (6)	0.3773 (8)	0.3664 (5)	0.0957 (11)	0.395 (6)
O5	1.0589 (4)	0.3329 (5)	0.3390 (3)	0.0957 (11)	0.605 (6)
O6	1.22955 (16)	0.3894 (2)	0.13260 (14)	0.0886 (8)	
O7	1.17901 (14)	0.49452 (17)	0.03706 (11)	0.0650 (5)	
O8A	0.3039 (6)	0.1518 (9)	0.1006 (6)	0.0706 (12)	0.395 (6)
O8	0.3337 (4)	0.1253 (5)	0.1125 (4)	0.0706 (12)	0.605 (6)
O9A	0.2177 (6)	0.0413 (8)	0.1356 (6)	0.0706 (12)	0.395 (6)
O9	0.2512 (4)	0.0092 (5)	0.1496 (3)	0.0706 (12)	0.605 (6)
O10	0.41754 (11)	0.24944 (14)	0.22274 (9)	0.0496 (4)	
O11	0.42811 (13)	0.42582 (16)	0.31111 (14)	0.0662 (5)	
O12	0.42006 (17)	0.3801 (2)	0.43080 (13)	0.0884 (8)	
O13	0.1432 (6)	0.1580 (6)	0.4551 (6)	0.0708 (7)	0.395 (6)
O13A	0.1624 (3)	0.1919 (4)	0.4605 (4)	0.0708 (7)	0.605 (6)
O14	0.0765 (6)	0.0945 (9)	0.3400 (4)	0.0708 (7)	0.395 (6)
O14A	0.0865 (4)	0.0942 (5)	0.3683 (3)	0.0708 (7)	0.605 (6)
N1	0.48222 (11)	0.27782 (13)	0.08706 (9)	0.0334 (4)	
H1N	0.4503 (15)	0.2601 (19)	0.1214 (12)	0.040*	
N2	0.77333 (12)	0.47437 (14)	0.33144 (10)	0.0385 (4)	
H2N	0.8096 (15)	0.483 (2)	0.2986 (13)	0.046*	
N3	0.89847 (15)	0.64836 (17)	0.09876 (12)	0.0513 (5)	
N4	1.02831 (16)	0.42310 (19)	0.32931 (13)	0.0576 (6)	
N5	1.17391 (15)	0.45175 (18)	0.10008 (13)	0.0532 (5)	
N6	0.27963 (15)	0.10320 (18)	0.15556 (12)	0.0529 (5)	
N7	0.40122 (14)	0.36817 (18)	0.35907 (13)	0.0545 (5)	
N8	0.14695 (19)	0.1484 (2)	0.39024 (16)	0.0692 (7)	
C1	0.46460 (15)	0.20288 (18)	0.01772 (12)	0.0409 (5)	
H1A	0.5179	0.1975	−0.0085	0.049*	
H1B	0.4493	0.1321	0.0351	0.049*	
C2	0.3856 (2)	0.2509 (2)	−0.03715 (16)	0.0621 (7)	
H2A	0.3894	0.2359	−0.0931	0.075*	
H2B	0.3280	0.2226	−0.0259	0.075*	
C3	0.3918 (2)	0.3664 (3)	−0.0214 (2)	0.0748 (9)	
H3A	0.4153	0.4027	−0.0646	0.090*	
H3B	0.3314	0.3951	−0.0186	0.090*	
C4	0.4532 (3)	0.3821 (2)	0.05422 (19)	0.0839 (11)	
H4A	0.4219	0.4208	0.0916	0.101*	
H4B	0.5063	0.4236	0.0459	0.101*	
C5	0.57653 (15)	0.2711 (2)	0.12893 (15)	0.0531 (6)	
H5A	0.6172	0.2871	0.0910	0.064*	
H5B	0.5889	0.1982	0.1477	0.064*	
C6	0.59780 (15)	0.3441 (2)	0.19836 (13)	0.0452 (5)	
H6A	0.5496	0.3785	0.2170	0.054*	
C7	0.68177 (14)	0.36208 (17)	0.23445 (11)	0.0363 (4)	
C8	0.69781 (13)	0.41772 (16)	0.31109 (11)	0.0343 (4)	
C9	0.64044 (16)	0.4112 (2)	0.36606 (13)	0.0447 (5)	
H9A	0.5864	0.3714	0.3540	0.054*	
C10	0.66114 (19)	0.4620 (2)	0.43778 (14)	0.0545 (6)	
H10A	0.6211	0.4578	0.4748	0.065*	
C11	0.7398 (2)	0.5186 (2)	0.45588 (13)	0.0536 (6)	
H11A	0.7553	0.5533	0.5055	0.064*	
C12	0.79475 (18)	0.52380 (19)	0.40122 (13)	0.0496 (6)	
H12A	0.8492	0.5630	0.4126	0.059*	
C13	0.76235 (13)	0.31890 (16)	0.20509 (11)	0.0343 (4)	
C14	0.81005 (15)	0.23621 (19)	0.24497 (12)	0.0434 (5)	
H14A	0.7913	0.2075	0.2906	0.052*	
C15	0.88497 (16)	0.19494 (19)	0.21895 (14)	0.0465 (5)	
H15A	0.9171	0.1386	0.2473	0.056*	
C16	0.91379 (14)	0.23425 (17)	0.15237 (13)	0.0395 (5)	
C17	0.86463 (15)	0.31561 (19)	0.11187 (13)	0.0436 (5)	
H17A	0.8822	0.3426	0.0653	0.052*	
C18	0.79042 (15)	0.35835 (18)	0.13792 (13)	0.0418 (5)	
H18A	0.7586	0.4150	0.1097	0.050*	
C19	0.99617 (17)	0.1905 (2)	0.12528 (17)	0.0578 (6)	
H19A	0.9904	0.1999	0.0679	0.087*	
H19B	1.0017	0.1156	0.1381	0.087*	
H19C	1.0500	0.2276	0.1522	0.087*	
C20	0.96436 (15)	0.57494 (16)	0.14058 (12)	0.0378 (4)	
C21	0.95360 (15)	0.53588 (16)	0.21766 (13)	0.0399 (5)	
C22	1.02623 (16)	0.46581 (18)	0.25059 (13)	0.0430 (5)	
C23	1.09501 (16)	0.43644 (18)	0.21281 (14)	0.0452 (5)	
H23A	1.1395	0.3880	0.2369	0.054*	
C24	1.09888 (15)	0.47827 (17)	0.13884 (13)	0.0414 (5)	
C25	1.03443 (15)	0.54831 (17)	0.10349 (12)	0.0389 (4)	
H25A	1.0386	0.5780	0.0535	0.047*	
C26	0.28089 (15)	0.15781 (17)	0.22957 (12)	0.0396 (5)	
C27	0.35152 (13)	0.23267 (16)	0.25547 (11)	0.0356 (4)	
C28	0.34105 (14)	0.28230 (18)	0.32932 (12)	0.0403 (5)	
C29	0.27778 (17)	0.25319 (19)	0.37352 (13)	0.0472 (6)	
H29A	0.2772	0.2848	0.4236	0.057*	
C30	0.21500 (17)	0.1777 (2)	0.34464 (14)	0.0492 (6)	
C31	0.21513 (16)	0.13026 (18)	0.27218 (14)	0.0453 (5)	
H31A	0.1706	0.0796	0.2521	0.054*	

### Atomic displacement parameters (Å^2^)

**Table:**

	*U*^11^	*U*^22^	*U*^33^	*U*^12^	*U*^13^	*U*^23^
O1	0.0933 (16)	0.0729 (14)	0.0937 (16)	0.0258 (12)	0.0423 (13)	0.0435 (12)
O2	0.0577 (12)	0.0972 (16)	0.0691 (12)	0.0240 (11)	0.0224 (10)	0.0111 (11)
O3	0.0602 (11)	0.0571 (10)	0.0597 (10)	−0.0013 (8)	0.0343 (9)	0.0068 (8)
O4A	0.126 (2)	0.110 (4)	0.0594 (17)	0.054 (2)	0.0387 (17)	0.0402 (14)
O4	0.126 (2)	0.110 (4)	0.0594 (17)	0.054 (2)	0.0387 (17)	0.0402 (14)
O5A	0.126 (2)	0.110 (4)	0.0594 (17)	0.054 (2)	0.0387 (17)	0.0402 (14)
O5	0.126 (2)	0.110 (4)	0.0594 (17)	0.054 (2)	0.0387 (17)	0.0402 (14)
O6	0.0714 (14)	0.116 (2)	0.0838 (15)	0.0450 (14)	0.0276 (12)	0.0182 (14)
O7	0.0716 (13)	0.0776 (13)	0.0537 (10)	0.0064 (10)	0.0325 (9)	−0.0010 (9)
O8A	0.077 (3)	0.083 (3)	0.058 (2)	−0.030 (2)	0.030 (2)	−0.0174 (18)
O8	0.077 (3)	0.083 (3)	0.058 (2)	−0.030 (2)	0.030 (2)	−0.0174 (18)
O9A	0.077 (3)	0.083 (3)	0.058 (2)	−0.030 (2)	0.030 (2)	−0.0174 (18)
O9	0.077 (3)	0.083 (3)	0.058 (2)	−0.030 (2)	0.030 (2)	−0.0174 (18)
O10	0.0442 (9)	0.0685 (11)	0.0417 (8)	−0.0085 (8)	0.0227 (7)	−0.0043 (7)
O11	0.0522 (11)	0.0573 (11)	0.0923 (15)	−0.0053 (9)	0.0221 (10)	−0.0129 (11)
O12	0.0864 (16)	0.1094 (19)	0.0609 (12)	0.0143 (14)	−0.0106 (11)	−0.0316 (13)
O13	0.0794 (16)	0.0789 (14)	0.0695 (15)	0.0012 (12)	0.0548 (18)	0.025 (2)
O13A	0.0794 (16)	0.0789 (14)	0.0695 (15)	0.0012 (12)	0.0548 (18)	0.025 (2)
O14	0.0794 (16)	0.0789 (14)	0.0695 (15)	0.0012 (12)	0.0548 (18)	0.025 (2)
O14A	0.0794 (16)	0.0789 (14)	0.0695 (15)	0.0012 (12)	0.0548 (18)	0.025 (2)
N1	0.0337 (8)	0.0413 (9)	0.0275 (7)	−0.0007 (7)	0.0115 (6)	−0.0027 (6)
N2	0.0427 (10)	0.0439 (9)	0.0312 (8)	−0.0069 (8)	0.0131 (7)	−0.0020 (7)
N3	0.0564 (12)	0.0488 (11)	0.0513 (11)	0.0073 (9)	0.0169 (10)	0.0010 (9)
N4	0.0621 (14)	0.0641 (14)	0.0504 (12)	0.0035 (11)	0.0199 (10)	0.0177 (10)
N5	0.0503 (12)	0.0612 (13)	0.0506 (11)	0.0047 (10)	0.0161 (10)	−0.0064 (10)
N6	0.0548 (12)	0.0615 (13)	0.0462 (10)	−0.0188 (10)	0.0193 (9)	−0.0072 (9)
N7	0.0426 (11)	0.0619 (13)	0.0572 (12)	0.0133 (10)	0.0038 (10)	−0.0164 (11)
N8	0.0687 (16)	0.0702 (15)	0.0831 (17)	0.0250 (13)	0.0529 (14)	0.0322 (13)
C1	0.0466 (12)	0.0449 (11)	0.0322 (9)	−0.0044 (9)	0.0094 (9)	−0.0069 (8)
C2	0.0660 (17)	0.0691 (17)	0.0445 (13)	−0.0034 (13)	−0.0085 (12)	0.0011 (12)
C3	0.0715 (19)	0.0651 (18)	0.077 (2)	0.0079 (15)	−0.0153 (16)	0.0112 (15)
C4	0.133 (3)	0.0419 (14)	0.0646 (17)	0.0206 (17)	−0.0165 (19)	−0.0034 (13)
C5	0.0363 (11)	0.0741 (17)	0.0498 (13)	0.0015 (11)	0.0102 (10)	−0.0227 (12)
C6	0.0361 (11)	0.0600 (14)	0.0414 (11)	−0.0013 (10)	0.0120 (9)	−0.0148 (10)
C7	0.0358 (10)	0.0438 (11)	0.0314 (9)	−0.0020 (8)	0.0118 (8)	−0.0038 (8)
C8	0.0334 (10)	0.0390 (10)	0.0323 (9)	0.0011 (8)	0.0107 (8)	0.0012 (8)
C9	0.0409 (11)	0.0579 (13)	0.0390 (10)	−0.0003 (10)	0.0173 (9)	0.0013 (10)
C10	0.0634 (16)	0.0695 (16)	0.0365 (11)	0.0107 (13)	0.0247 (11)	0.0009 (11)
C11	0.0757 (18)	0.0528 (14)	0.0334 (10)	0.0053 (12)	0.0124 (11)	−0.0094 (10)
C12	0.0606 (15)	0.0464 (12)	0.0405 (11)	−0.0087 (11)	0.0057 (10)	−0.0055 (9)
C13	0.0305 (9)	0.0427 (11)	0.0307 (9)	−0.0043 (8)	0.0080 (7)	−0.0061 (8)
C14	0.0442 (12)	0.0559 (13)	0.0307 (9)	0.0012 (10)	0.0081 (9)	0.0037 (9)
C15	0.0438 (12)	0.0508 (13)	0.0441 (11)	0.0097 (10)	0.0055 (9)	0.0043 (10)
C16	0.0326 (10)	0.0449 (11)	0.0414 (10)	−0.0023 (8)	0.0073 (8)	−0.0108 (9)
C17	0.0431 (12)	0.0525 (13)	0.0393 (10)	−0.0011 (10)	0.0185 (9)	0.0025 (9)
C18	0.0380 (11)	0.0482 (12)	0.0419 (11)	0.0028 (9)	0.0142 (9)	0.0072 (9)
C19	0.0445 (13)	0.0637 (16)	0.0689 (16)	0.0064 (12)	0.0195 (12)	−0.0130 (13)
C20	0.0415 (11)	0.0345 (10)	0.0388 (10)	−0.0045 (8)	0.0106 (8)	−0.0029 (8)
C21	0.0454 (11)	0.0355 (10)	0.0415 (10)	−0.0101 (9)	0.0150 (9)	−0.0034 (8)
C22	0.0504 (13)	0.0427 (11)	0.0371 (10)	−0.0085 (9)	0.0110 (9)	0.0032 (9)
C23	0.0468 (12)	0.0443 (12)	0.0436 (11)	−0.0016 (9)	0.0057 (10)	0.0007 (9)
C24	0.0420 (11)	0.0445 (11)	0.0393 (10)	−0.0032 (9)	0.0119 (9)	−0.0087 (9)
C25	0.0452 (11)	0.0396 (11)	0.0333 (9)	−0.0069 (9)	0.0110 (9)	−0.0046 (8)
C26	0.0403 (11)	0.0445 (11)	0.0371 (10)	0.0021 (9)	0.0149 (9)	0.0050 (8)
C27	0.0339 (10)	0.0432 (11)	0.0316 (9)	0.0058 (8)	0.0115 (8)	0.0052 (8)
C28	0.0385 (11)	0.0459 (11)	0.0380 (10)	0.0119 (9)	0.0109 (8)	0.0003 (9)
C29	0.0532 (13)	0.0560 (13)	0.0369 (10)	0.0233 (11)	0.0205 (10)	0.0074 (10)
C30	0.0509 (13)	0.0522 (13)	0.0531 (13)	0.0174 (11)	0.0328 (11)	0.0201 (11)
C31	0.0415 (11)	0.0444 (12)	0.0540 (13)	0.0032 (9)	0.0198 (10)	0.0116 (10)

### Geometric parameters (Å, °)

**Table:**

O1—N3	1.220 (3)	C5—H5A	0.9900
O2—N3	1.211 (3)	C5—H5B	0.9900
O3—C21	1.247 (3)	C6—C7	1.325 (3)
O4A—N4	1.280 (11)	C6—H6A	0.9500
O4—N4	1.166 (7)	C7—C8	1.475 (3)
O5A—N4	1.242 (9)	C7—C13	1.498 (3)
O5—N4	1.238 (6)	C8—C9	1.389 (3)
O6—N5	1.216 (3)	C9—C10	1.375 (3)
O7—N5	1.225 (3)	C9—H9A	0.9500
O8A—N6	1.235 (10)	C10—C11	1.374 (4)
O8—N6	1.225 (6)	C10—H10A	0.9500
O9A—N6	1.224 (10)	C11—C12	1.358 (4)
O9—N6	1.270 (6)	C11—H11A	0.9500
O10—C27	1.244 (2)	C12—H12A	0.9500
O11—N7	1.224 (3)	C13—C14	1.386 (3)
O12—N7	1.222 (3)	C13—C18	1.389 (3)
O13—N8	1.130 (10)	C14—C15	1.388 (3)
O13A—N8	1.309 (7)	C14—H14A	0.9500
O14—N8	1.419 (12)	C15—C16	1.385 (3)
O14A—N8	1.152 (8)	C15—H15A	0.9500
N1—C5	1.475 (3)	C16—C17	1.387 (3)
N1—C4	1.480 (3)	C16—C19	1.506 (3)
N1—C1	1.511 (3)	C17—C18	1.386 (3)
N1—H1N	0.854 (16)	C17—H17A	0.9500
N2—C8	1.340 (3)	C18—H18A	0.9500
N2—C12	1.340 (3)	C19—H19A	0.9800
N2—H2N	0.858 (17)	C19—H19B	0.9800
N3—C20	1.457 (3)	C19—H19C	0.9800
N4—C22	1.451 (3)	C20—C25	1.368 (3)
N5—C24	1.448 (3)	C20—C21	1.448 (3)
N6—C26	1.445 (3)	C21—C22	1.447 (3)
N7—C28	1.454 (3)	C22—C23	1.367 (3)
N8—C30	1.444 (3)	C23—C24	1.386 (3)
C1—C2	1.509 (3)	C23—H23A	0.9500
C1—H1A	0.9900	C24—C25	1.378 (3)
C1—H1B	0.9900	C25—H25A	0.9500
C2—C3	1.497 (4)	C26—C31	1.376 (3)
C2—H2A	0.9900	C26—C27	1.439 (3)
C2—H2B	0.9900	C27—C28	1.449 (3)
C3—C4	1.467 (4)	C28—C29	1.369 (3)
C3—H3A	0.9900	C29—C30	1.378 (4)
C3—H3B	0.9900	C29—H29A	0.9500
C4—H4A	0.9900	C30—C31	1.382 (3)
C4—H4B	0.9900	C31—H31A	0.9500
C5—C6	1.501 (3)		
			
C5—N1—C4	115.6 (2)	C8—C7—C13	117.49 (17)
C5—N1—C1	111.42 (16)	N2—C8—C9	117.15 (19)
C4—N1—C1	105.67 (18)	N2—C8—C7	119.24 (17)
C5—N1—H1N	105.1 (16)	C9—C8—C7	123.55 (19)
C4—N1—H1N	109.6 (17)	C10—C9—C8	120.6 (2)
C1—N1—H1N	109.5 (16)	C10—C9—H9A	119.7
C8—N2—C12	123.00 (19)	C8—C9—H9A	119.7
C8—N2—H2N	120.3 (17)	C11—C10—C9	119.9 (2)
C12—N2—H2N	116.6 (18)	C11—C10—H10A	120.0
O2—N3—O1	122.9 (2)	C9—C10—H10A	120.0
O2—N3—C20	119.6 (2)	C12—C11—C10	118.4 (2)
O1—N3—C20	117.4 (2)	C12—C11—H11A	120.8
O4—N4—O5	117.2 (4)	C10—C11—H11A	120.8
O4—N4—O5A	106.4 (6)	N2—C12—C11	120.9 (2)
O5—N4—O4A	131.5 (5)	N2—C12—H12A	119.5
O4—N4—C22	126.0 (3)	C11—C12—H12A	119.5
O5—N4—C22	114.8 (3)	C14—C13—C18	118.50 (19)
O5A—N4—C22	122.3 (4)	C14—C13—C7	119.56 (18)
O4A—N4—C22	112.1 (4)	C18—C13—C7	121.93 (19)
O6—N5—O7	123.3 (2)	C13—C14—C15	120.6 (2)
O6—N5—C24	118.2 (2)	C13—C14—H14A	119.7
O7—N5—C24	118.5 (2)	C15—C14—H14A	119.7
O9A—N6—O8	122.3 (4)	C16—C15—C14	121.3 (2)
O9A—N6—O8A	115.1 (8)	C16—C15—H15A	119.4
O8—N6—O9	114.8 (5)	C14—C15—H15A	119.4
O8A—N6—O9	123.6 (5)	C15—C16—C17	117.8 (2)
O9A—N6—C26	116.6 (5)	C15—C16—C19	121.0 (2)
O8—N6—C26	120.8 (4)	C17—C16—C19	121.2 (2)
O8A—N6—C26	117.9 (6)	C18—C17—C16	121.5 (2)
O9—N6—C26	118.5 (3)	C18—C17—H17A	119.3
O12—N7—O11	123.8 (3)	C16—C17—H17A	119.3
O12—N7—C28	117.8 (3)	C17—C18—C13	120.4 (2)
O11—N7—C28	118.4 (2)	C17—C18—H18A	119.8
O13—N8—O14A	102.2 (4)	C13—C18—H18A	119.8
O14A—N8—O13A	123.8 (3)	C16—C19—H19A	109.5
O13—N8—O14	119.2 (5)	C16—C19—H19B	109.5
O13A—N8—O14	139.1 (4)	H19A—C19—H19B	109.5
O13—N8—C30	132.0 (5)	C16—C19—H19C	109.5
O14A—N8—C30	125.0 (3)	H19A—C19—H19C	109.5
O13A—N8—C30	111.2 (3)	H19B—C19—H19C	109.5
O14—N8—C30	108.8 (3)	C25—C20—C21	124.0 (2)
C2—C1—N1	104.05 (19)	C25—C20—N3	116.41 (19)
C2—C1—H1A	110.9	C21—C20—N3	119.58 (19)
N1—C1—H1A	110.9	O3—C21—C22	124.4 (2)
C2—C1—H1B	110.9	O3—C21—C20	124.0 (2)
N1—C1—H1B	110.9	C22—C21—C20	111.67 (19)
H1A—C1—H1B	109.0	C23—C22—C21	124.6 (2)
C3—C2—C1	105.7 (2)	C23—C22—N4	116.4 (2)
C3—C2—H2A	110.6	C21—C22—N4	119.0 (2)
C1—C2—H2A	110.6	C22—C23—C24	119.1 (2)
C3—C2—H2B	110.6	C22—C23—H23A	120.5
C1—C2—H2B	110.6	C24—C23—H23A	120.5
H2A—C2—H2B	108.7	C25—C24—C23	120.7 (2)
C4—C3—C2	107.6 (2)	C25—C24—N5	119.7 (2)
C4—C3—H3A	110.2	C23—C24—N5	119.5 (2)
C2—C3—H3A	110.2	C20—C25—C24	119.84 (19)
C4—C3—H3B	110.2	C20—C25—H25A	120.1
C2—C3—H3B	110.2	C24—C25—H25A	120.1
H3A—C3—H3B	108.5	C31—C26—C27	124.6 (2)
C3—C4—N1	108.1 (2)	C31—C26—N6	116.4 (2)
C3—C4—H4A	110.1	C27—C26—N6	118.99 (18)
N1—C4—H4A	110.1	O10—C27—C26	125.50 (19)
C3—C4—H4B	110.1	O10—C27—C28	122.6 (2)
N1—C4—H4B	110.1	C26—C27—C28	111.73 (18)
H4A—C4—H4B	108.4	C29—C28—C27	124.0 (2)
N1—C5—C6	113.65 (19)	C29—C28—N7	117.4 (2)
N1—C5—H5A	108.8	C27—C28—N7	118.53 (19)
C6—C5—H5A	108.8	C28—C29—C30	119.4 (2)
N1—C5—H5B	108.8	C28—C29—H29A	120.3
C6—C5—H5B	108.8	C30—C29—H29A	120.3
H5A—C5—H5B	107.7	C29—C30—C31	121.2 (2)
C7—C6—C5	122.0 (2)	C29—C30—N8	119.6 (2)
C7—C6—H6A	119.0	C31—C30—N8	119.2 (3)
C5—C6—H6A	119.0	C26—C31—C30	118.8 (2)
C6—C7—C8	119.40 (18)	C26—C31—H31A	120.6
C6—C7—C13	122.81 (18)	C30—C31—H31A	120.6
			
C5—N1—C1—C2	−155.6 (2)	O4A—N4—C22—C23	158.7 (12)
C4—N1—C1—C2	−29.3 (3)	O4—N4—C22—C21	−17.2 (10)
N1—C1—C2—C3	27.9 (3)	O5—N4—C22—C21	146.1 (4)
C1—C2—C3—C4	−16.3 (4)	O5A—N4—C22—C21	−167.7 (7)
C2—C3—C4—N1	−2.2 (4)	O4A—N4—C22—C21	−20.9 (13)
C5—N1—C4—C3	143.4 (3)	C21—C22—C23—C24	2.3 (4)
C1—N1—C4—C3	19.7 (4)	N4—C22—C23—C24	−177.2 (2)
C4—N1—C5—C6	59.3 (3)	C22—C23—C24—C25	−0.1 (3)
C1—N1—C5—C6	179.9 (2)	C22—C23—C24—N5	176.9 (2)
N1—C5—C6—C7	−168.2 (2)	O6—N5—C24—C25	−179.8 (2)
C5—C6—C7—C8	−168.2 (2)	O7—N5—C24—C25	1.5 (3)
C5—C6—C7—C13	5.4 (4)	O6—N5—C24—C23	3.1 (4)
C12—N2—C8—C9	−0.1 (3)	O7—N5—C24—C23	−175.6 (2)
C12—N2—C8—C7	−177.3 (2)	C21—C20—C25—C24	1.7 (3)
C6—C7—C8—N2	−151.8 (2)	N3—C20—C25—C24	−179.20 (19)
C13—C7—C8—N2	34.2 (3)	C23—C24—C25—C20	−1.8 (3)
C6—C7—C8—C9	31.2 (3)	N5—C24—C25—C20	−178.9 (2)
C13—C7—C8—C9	−142.8 (2)	O9A—N6—C26—C31	3.5 (6)
N2—C8—C9—C10	0.4 (3)	O8—N6—C26—C31	177.4 (4)
C7—C8—C9—C10	177.5 (2)	O8A—N6—C26—C31	146.8 (5)
C8—C9—C10—C11	−0.8 (4)	O9—N6—C26—C31	−31.1 (4)
C9—C10—C11—C12	0.8 (4)	O9A—N6—C26—C27	−179.2 (5)
C8—N2—C12—C11	0.1 (4)	O8—N6—C26—C27	−5.2 (5)
C10—C11—C12—N2	−0.5 (4)	O8A—N6—C26—C27	−35.9 (6)
C6—C7—C13—C14	−105.8 (3)	O9—N6—C26—C27	146.2 (4)
C8—C7—C13—C14	67.9 (3)	C31—C26—C27—O10	170.8 (2)
C6—C7—C13—C18	73.2 (3)	N6—C26—C27—O10	−6.3 (3)
C8—C7—C13—C18	−113.0 (2)	C31—C26—C27—C28	−4.7 (3)
C18—C13—C14—C15	1.0 (3)	N6—C26—C27—C28	178.24 (19)
C7—C13—C14—C15	−179.9 (2)	O10—C27—C28—C29	−168.8 (2)
C13—C14—C15—C16	−0.5 (4)	C26—C27—C28—C29	6.9 (3)
C14—C15—C16—C17	−0.7 (3)	O10—C27—C28—N7	11.0 (3)
C14—C15—C16—C19	178.8 (2)	C26—C27—C28—N7	−173.36 (18)
C15—C16—C17—C18	1.6 (3)	O12—N7—C28—C29	30.3 (3)
C19—C16—C17—C18	−178.0 (2)	O11—N7—C28—C29	−147.8 (2)
C16—C17—C18—C13	−1.2 (3)	O12—N7—C28—C27	−149.5 (2)
C14—C13—C18—C17	−0.1 (3)	O11—N7—C28—C27	32.4 (3)
C7—C13—C18—C17	−179.2 (2)	C27—C28—C29—C30	−5.1 (3)
O2—N3—C20—C25	157.6 (2)	N7—C28—C29—C30	175.1 (2)
O1—N3—C20—C25	−21.4 (3)	C28—C29—C30—C31	0.5 (3)
O2—N3—C20—C21	−23.2 (3)	C28—C29—C30—N8	−178.6 (2)
O1—N3—C20—C21	157.8 (2)	O13—N8—C30—C29	−19.7 (7)
C25—C20—C21—O3	179.8 (2)	O14A—N8—C30—C29	172.0 (4)
N3—C20—C21—O3	0.7 (3)	O13A—N8—C30—C29	−7.8 (4)
C25—C20—C21—C22	0.3 (3)	O14—N8—C30—C29	163.1 (4)
N3—C20—C21—C22	−178.81 (19)	O13—N8—C30—C31	161.1 (6)
O3—C21—C22—C23	178.2 (2)	O14A—N8—C30—C31	−7.1 (5)
C20—C21—C22—C23	−2.3 (3)	O13A—N8—C30—C31	173.1 (3)
O3—C21—C22—N4	−2.3 (3)	O14—N8—C30—C31	−16.0 (5)
C20—C21—C22—N4	177.22 (19)	C27—C26—C31—C30	0.8 (3)
O4—N4—C22—C23	162.4 (10)	N6—C26—C31—C30	177.9 (2)
O5—N4—C22—C23	−34.3 (5)	C29—C30—C31—C26	1.6 (3)
O5A—N4—C22—C23	11.9 (7)	N8—C30—C31—C26	−179.3 (2)

### Hydrogen-bond geometry (Å, °)

**Table:**

*D*—H···*A*	*D*—H	H···*A*	*D*···*A*	*D*—H···*A*
N1—H1N···O10	0.85 (2)	1.89 (2)	2.701 (2)	157 (2)
N1—H1N···O8	0.85 (2)	2.44 (2)	3.053 (7)	129 (2)
N1—H1N···O8A	0.85 (2)	2.57 (2)	3.173 (10)	128 (2)
N2—H2N···O3	0.86 (2)	1.85 (2)	2.648 (2)	153 (2)
C31—H31A···O11^i^	0.95	2.58	3.520 (3)	170.
C9—H9A···O11	0.95	2.47	3.174 (3)	131.
C5—H5B···O3^ii^	0.99	2.44	3.362 (4)	154.
C5—H5A···O13A^iii^	0.99	2.47	3.399 (6)	157.
C4—H4A···O14A^iv^	0.99	2.33	3.116 (7)	136.

Symmetry codes: (i) −*x*+1/2, *y*−1/2, −*z*+1/2; (ii) −*x*+3/2, *y*−1/2, −*z*+1/2; (iii) *x*+1/2, −*y*+1/2, *z*−1/2; (iv) −*x*+1/2, *y*+1/2, −*z*+1/2.
